# MiR-222-3p in Platelets Serves as a Distinguishing Marker for Early Recognition of Kawasaki Disease

**DOI:** 10.3389/fped.2019.00237

**Published:** 2019-06-28

**Authors:** Bo Wang, Li-nong Wang, Fang-fang Cheng, Hai-tao Lv, Ling Sun, Dong-kai Wei, Yu Pu, Jie Wu, Yuan-yuan Hou, Bin Wen, Xia-ping Xu, Wen-hua Yan

**Affiliations:** ^1^Internal Medicine-Cardiovascular Department, Children's Hospital of Soochow University, Suzhou, China; ^2^QIAGEN (Suzhou) Translational Medicine Co., Ltd., Suzhou, China

**Keywords:** Kawasaki disease, other febrile illness, miRNAs, platelets, miR-222-3p

## Abstract

Kawasaki disease (KD) is an acute vasculitis, which leads to 20% of sufferers developing coronary artery aneurysm in children if not appropriately treated. Therefore, the early diagnosis of KD is essential for alleviating the risk of developing heart disease. MicroRNAs (miRNAs) are a large class of small non-coding RNAs which post-transcriptionally regulate gene expression and have been shown to play critical roles in numerous biological processes and diseases. In this study, we used high-throughput miRNA sequencing and found dozens of miRNAs are highly expressed in platelets. By comparing the miRNA expression profile of platelets of acute KD patients and other febrile patients, miR-222-3p is validated to be significantly upregulated in platelets of acute KD patients. Furthermore, KEGG pathway analysis shows that targets of miR-222-3p are enriched in immune-related signaling pathways. Our study uncovers the potential of miR-222-3p in platelets as biomarker for early diagnosis of Kawasaki disease.

## Introduction

Kawasaki disease (KD) is also known as mucocutaneous lymph node syndrome (1), which is a systemic vasculitis and its etiology remains obscure. Asian children and those younger than 5 years are more prone to be afflicted with Kawasaki disease (2). Approximately 15–25% cases of Kawasaki disease children may develop coronary artery abnormalities if not treated appropriately (3). It turns out to be the leading cause of pediatric acquired heart disease and increases the risk of myocardial infarction (4).

According to previous studies, intravenous immunoglobulin (IVIG) and aspirin can significantly reduce the incidence of coronary artery lesions (CALs) to ~5% if treated in the first 10 days of KD (5, 6). However, the diagnosis of KD can be very challenging. Kawasaki disease is presented by prolonged fever for at least 5 days and coupled with at least four of the following clinical criteria: (1) bilateral non-exudative conjunctival injection; (2) changes in the mucosa of the oropharynx, including injected pharynx, dry fissured lips and strawberry tongue; (3) polymorphous exanthema; (4) changes of the peripheral extremities such as indurative oedema or erythema of hands and feet in the acute phase, and later membranous desquamation starting around the nail bed; (5) cervical lymphadenopathy >1.5 cm usually unilateral (7–11). Children with inadequate diagnostic criteria are classified as incomplete KD or atypical KD patients (8, 12), who are easily misdiagnosed and their treatment may be subsequently delayed, which greatly increases the risk of CALs. Thus, the identification of reliable biomarkers may facilitate early diagnosis and effective treatment of KD.

Some attempts have been made to identify protein biomarkers for KD. A study indicated that fibrinogen-related plasma protein (fibrinogen, alpha-1-antitrypsin, clusterin, and CD5L) levels are highly elevated during acute KD (13), among which plasma clusterin, also known as apolipoprotein J, has been recognized as a prognostic biomarker for CAL sequelae in KD (14). Besides, N-terminal pro-BNP (NTpro-BNP) was found to be elevated in the acute phase of KD (13, 15) and possess diagnostic utility and predictive value (16, 17). Kim et al. reported serum cardiac troponin I (cTnI) and creatinine kinase (CK)-MB as two biomarkers, occurring at higher level in KD patients in comparison to age-matched, non-KD control patients (18). Furthermore, nitric oxide synthases (iNOS) in neutrophils have also been identified as a promising biomarker, and have been found at higher expression levels during acute KD (19). Another important finding is that CXCL10 (IP-10) levels were significantly increased in KD patients, and there was IP-10 receptor CXCR3 activation in T cells of the acute KD cohort (20), providing insight into the use of cytokines as biomarkers for KD (21). Moreover, Yayoi et al. proved the potential of LRG1 as a biomarker to facilitate KD diagnosis by mass spectrometry (MS) (22).

Although various biomarkers have been reported, the consistency between different studies remains challenged. Besides, the low sensitivity to quantify low-abundant proteins and antibody-based detection limits the utility of protein biomarkers (23). Therefore, much easier detected and more delicate biomarkers are necessary.

Recent studies on cancer have revealed the potential importance of platelets in biomarker assessment from blood samples (24). Circulating platelets were reported to crosstalk with various cells such as leukocyte, endothelial cell et al. and molecules like ATP, thus serving as active media of intracellular communication. Furthermore, the transcriptome of platelets is swift altered responding to extracellular queues (25). Apart from mRNAs, platelet transcriptome contains various non-coding RNAs, including microRNAs and circular RNAs. These non-coding RNAs involve in multifarious biological processes, including vascular homeostasis, inflammation and contribute to platelet function (14).

To date, whether these non-coding RNAs, especially miRNAs in platelets have the potential as diagnostic biomarkers is still not clear. MicroRNAs (miRNAs) are a class of small RNA transcripts ranging from 18 to 25 nucleotides which post-transcriptionally regulate gene expression through destabilizing mRNA and/or translation inhibition. Previous studies have demonstrated that miRNAs play critical roles in numerous biological processes and diseases such as tumorigenesis (26–30), immune responses (31–34), differentiation (35–39), and apoptosis (40, 41). Furthermore, miRNA profiles are specific to various physiological and pathological conditions (42). Additionally, miRNA profiling has been shown to be more accurate than mRNA expression profiling in characterizing the difference of multiple human cancers (43), which postulates the possibility of platelets miRNAs as predictive biomarkers (44). In the current study, we investigated the potential of platelet miRNAs as biomarker for early diagnosis of Kawasaki disease and identified miR-222-3p as a distinguishing marker.

## Methods

### Platelets Isolation

This study was carried out in accordance with the principles of the Basel Declaration and recommendations of guidelines for good clinical practice (GCP). The protocol (No.2018LW001) was approved by Children's Hospital of Soochow University ethics committee. Peripheral blood from KD patients and other febrile controls was obtained at initial diagnosis from Children's Hospital of Soochow University, Suzhou. Platelets were isolated from plasma at room temperature. In brief, a total of 1 ml peripheral whole blood was collected from the patients, anticoagulated with EDTA in purple-cap BD Vacutainers. The blood was centrifuged at 120 g for 20 min to collect platelet-rich plasma. The platelet-rich plasma was further centrifuged at 360 g for 20 min, after which the platelet pellet was washed twice in PBS (Gibco). The isolated platelet pellets were snap-frozen at −80°C for future use.

### RNA Isolation

The mirVana™ miRNA isolation kit (P/N: AM1560, Applied Biosystems) was chosen for the isolation of platelet miRNAs, and used according to the manufacturer's instructions with some modifications. Briefly, the platelet pellet was lysed and vortexed to obtain a homogenous lysate. A tenth volume of miRNA homogenate additive was added to the lysate and the sample was mixed rigorously by vortex before incubation on ice for 10 min. The mix was purified with acid-phenol: chloroform, followed by filter through solid-phase cartridge. The flow-through was discarded and the column was subjected to washing steps. 100μl of nuclease-free water, preheated to 95°C, was used to elute platelet miRNA and centrifuged at 11,000 g for 45 s. The RNA was quantified with Nanodrop spectrophotometer (ND1000). RNA sample quality was evaluated by Agilent 2100 Bioanalyzer (Agilent Technologies).

### Small RNA Library Construction and Sequencing

All small RNA libraries were prepared using NEBNext Multiplex Small RNA Library Prep Set (NEW ENGLAND Biolabs). Briefly, 100 ng of total RNA was prepared from each sample to ligate diluted 3' SR adaptor directly. Subsequently excessed 3′ SR adaptors were hybridized with SR RT primer to prevent adaptor-dimer formation, followed by ligation of 5′ SR adaptor with T4 RNA ligase and reverse transcription to generate single-strand cDNA. To enrich the products for sequencing, 15-cycle PCR amplification was performed on the first cDNA strands using Illumina compatible multiplexed primer sets. The resulting library was subjected to size selection and purification with 10% PAGE gel (Solarbio). The concentration and quality of each small RNA library was examined by Qubit 2.0 (Invitrogen) and DNA High Sensitivity Chip (Agilent). High quality libraries with size between 150 and 160bp were pooled in equimolar concentration and sequenced for SE50 on Hiseq 2500 (Illumina).

### Quantitative Real-Time PCR (qRT-PCR) Validation of miRNAs

The cDNA was synthesized in a 25 μl volume containing 50 ng total RNA, 5 μl 5 × PAP/RT buffer, 1 μl RTase mix and 1 μl 2.5 U/μl Poly A Polymerase included in the All-in-One™ miRNA First-Strand cDNA Synthesis kit (GeneCopoeia). RT reactions were incubated at 37°C for 1 h, followed by 85°C for 5 min to inactivate reverse transcriptase mix and placed on ice. The resultant cDNAs were diluted to 1 ng/μl before use. MiRNA qPCR primers were designed based on mature miRNA sequences from miRBase v20. For has-miR-222-3p the primer sequence is AGCTACATCTGGCTACTGGGTAA and for C.elegans miR-39-3p the primer sequence is: TCACCGGGTGTAAATCAGCTTGAA. Quantitative real-time PCR reactions were conducted on the 7500 Real-Time PCR system (Applied Biosystems) using the All-in-OneTM miRNA qRT-PCR Detection kit (GeneCopoeia).

### Bioinformatics

MiRNA sequencing reads were matched to the miRBase miRNA hairpin precursors by mapper.pl provided by miRDeep2, after trimming the adapter sequence with fastx_clipper from fastx toolkit. CPM was obtained with the raw read count of each miRNA normalizing to library size of corresponding sample. R 3.5.1 was used for the generation of venn diagram, heatmap and violin plot. Python 3.5.5 was used to build random forest classifier model and generate receiver operating characteristic (ROC) curve and histogram.

## Results

### Sets of miRNAs Are Differentially Expressed in Platelets of Kawasaki Disease and Other Febrile Patients

To identify candidate miRNAs differentiating Kawasaki disease (KD) and other febrile illness (OFI), we enrolled 32 pediatric patients, including 16 children diagnosed with KD and 16 diagnosed with pneumonia, Bronchitis et al. which were grouped into OFI (Supplementary Table 1). Patients included in KD and OFI were matched for age and prolonged fever time (Supplementary Figure 1). Total RNA was extracted from platelets after elaborating isolation of platelets from whole blood. The platelet RNA was subjected to small RNA sequencing library construction and sequenced. Count per million (CPM) was used for expression level quantification. MiRNAs with CPM higher than 100 in at least half samples were considered expressed for further analysis (Supplementary Table 2).

**Figure 1:**
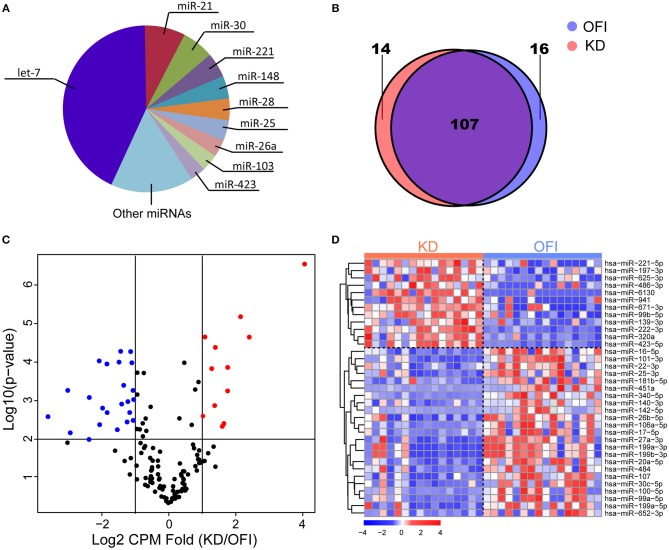
MiRNA expression profile of platelets of Kawasaki disease and other febrile patients. **(A)** Ten most abundant miRNA families expressed in platelets of KD samples. **(B)** Patients with KD or OFI share majority of expressed miRNAs in platelets. **(C)** Volcano plot of expressed miRNAs. **(D)** Heatmap of 35 miRNAs differentially expressed in KD compared with other febrile patients.

The most abundant microRNAs in platelets of KD patients are members of the let-7 microRNA family, which represented 42.84% of the platelet microRNA content (Figure 1A), consistent with previous identified miRNA profile in human platelets (45–47). The data pertaining to the 10 most abundant platelet microRNA families are compiled (Supplementary Table 3) and show that individual microRNAs, such as miR-21-5p, miR-30a/d-5p, miR-92a-3p, miR-103a-3p, miR-148a-3p, miR-26a-5p, miR-222-3p, and miR-151a-3p also accounted for an important proportion of human platelet microRNAs. 123 and 121 miRNAs were detected in platelets of OFI and KD patients, respectively, and the majority of miRNAs (78.1%) were both expressed in different disease states (Figure 1B), including key miRNAs in innate immune responses such as miR-146a and miR-155 (48). Among the expressed miRNAs in either KD or OFI patients' platelets, volcano plot showed that 35 miRNAs were differentially regulated with average CPM change for more than 2 folds and *p*-value < 0.01 (Figures 1C,D). 12 miRNAs were upregulated in KD patients' platelets while 23 miRNAs were downregulated.

### MiR-222-3p Is Identified as a Distinguishing Marker in KD and OFI Patients

**Figure 2:**
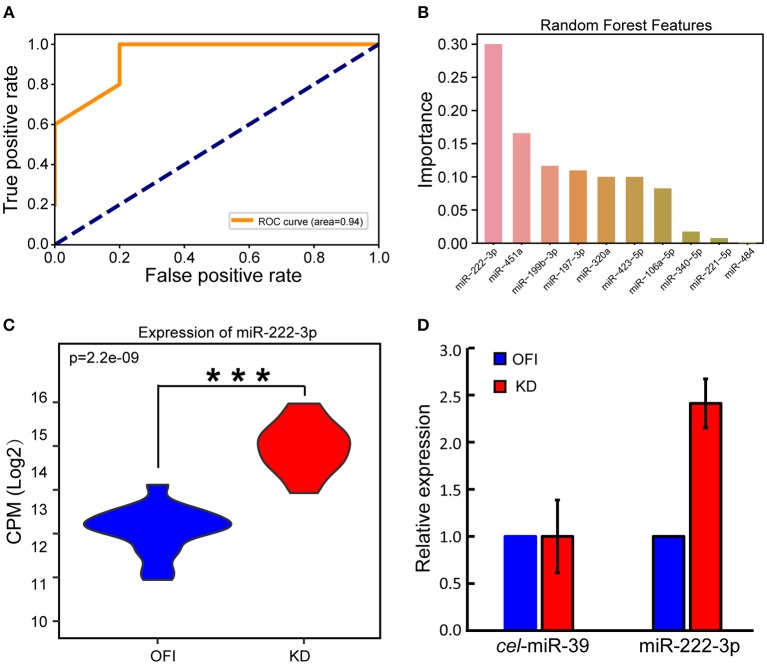
MiR-222-3p was identified as a distinguishing marker in KD and OFI samples. **(A)** Receiver operating characteristic (ROC) curve of RFC model on test set. **(B)** Relative rank of miRNAs used as decision node in the RFC model. **(C)** CPM distribution of miR-222-3p by small RNA sequencing. **(D)** qRT-PCR validation of miR-222-3p expression. C.elegans miR-39-3p was used as control. ***P < 0.001.

We reasoned that these differentially expressed miRNAs may largely contribute to the distinguished bio-signatures of KD and OFI patients, and thus may play important roles in the early diagnosis of Kawasaki disease. A random forest classifier (RFC) model was built with a training set containing 22 samples (11 KD and 11 OFI) using differentially expressed 35 miRNAs as features. Parameters were optimized by Bayesian Optimization (49) and used to train the model. Furthermore, we examined the generalization ability of the model by interrogating the remaining 10 samples (5 KD and 5 OFI). The receiver operating characteristic (ROC) curve was generated by comparing the predicted result with true sample class and the area under curve (AUC) reached 0.94 (Figure 2A), which suggested the high quality of this model in distinguishing KD from OFI samples. These data showed that the differentially expressed platelet miRNAs hold great potential in distinguishing KD patients from other febrile illness patients. To determine the most important miRNA in differentiating KD and OFI patients, the relative rank (i.e., depth) of miRNAs was used as decision node in the RFC model. As a result, miR-222-3p was shown as the miRNA feature with greatest importance (Figure 2B). MiR-222-3p shares identical seed sequence “GCUACAU” with miR-221-3p, both of which belong to miR-221 family of miRNAs, are highly expressed in platelets of KD patients (Figure 1A), which denotes it as a potent distinguishing marker in KD and OFI.

### MiR-222-3p Is Validated to Be Upregulated in Platelets of KD Patients

With the high throughput miRNA sequencing data, we found that miR-222-3p expression was significantly upregulated in KD patients comparing with OFI patients (Figure 2C). Quantitative real time PCR (qRT-PCR) was performed to validate the expression change of miR-222-3p in KD patients. Due to the limited amount of platelet RNA available from patients, we only performed qPCR on 6 samples with platelet RNA remained (3 KD and 3 OFI). With C.elegans miR-39-3p as exogenous miRNA normalization control as suggested by Nicholas et al. (47), miR-222-3p was upregulated for 2.41 fold (Figure 2D), which was consistent with the small RNA sequencing data. These data demonstrate that miR-222-3p is upregulated in platelets of KD patients, which may act as a potential biomarker for the diagnosis of Kawasaki disease.

### KEGG Pathway Enrichment of Predicted Target Genes of miR-222-3p

**Figure 3:**
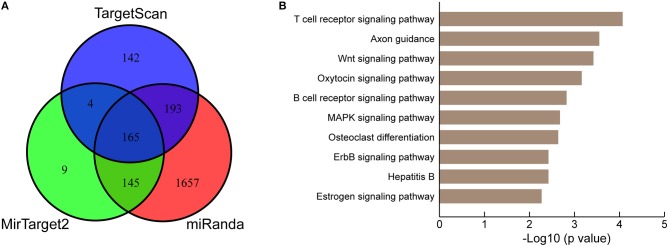
KEGG pathway analysis of miR-222-3p predicted targets. **(A)** Three miRNA target prediction tools are used for identifying authentic targets of miR-222-3p. **(B)** KEGG pathways enriched by common miR-222-3p predicted targets.

To further understand the biological significance of the upregulation of miR-222-3p in platelets of KD patients, we conducted KEGG pathway enrichment analysis of predicted miR-222-3p target genes. Three target prediction tools were chosen to identify authentic target genes of miR-222-3p, including TargetScan (50), miRanda (51) and MirTarget2 (52). A total of 165 common target genes of hsa-miR-222-3p were identified by comparing three sets of predicted target genes (Figure 3A). DAVID (53) was used for KEGG pathway enrichment analysis and the top 10 pathways were listed (Figure 3B). Surprisingly, the predicted target genes of miR-222-3p were most enriched in the T cell receptor signaling pathway, as well as B cell receptor signaling pathway, suggesting the involvement of platelet miRNAs in immune dysfunction. Consistently, KD is characterized with down-regulation of T cell receptor and B cell receptor signaling pathways by several studies (54–56).

## Discussion

Due to the long-lasting and detrimental coronary effects that Kawasaki disease may cause (11), accurate early diagnosis is necessitated for early recognition of the disease. To our knowledge, this is the first study showing potential of implementing platelet miRNAs in clinical practice for the diagnosis of Kawasaki disease. Here we show that human platelets express dozens of miRNAs, including miRNA families reported previously, such as let-7, miR-21, miR-25, miR-203 et al. (45). We further identify 35 miRNAs differentially expressed in platelets of KD patients and other febrile patients, among which miR-222-3p was validated to be upregulated in KD platelets. KEGG pathway analysis revealed that the targets of miR-222-3p were enriched in T-cell receptor pathway, indicating the crosstalk of miRNA between immune pathways. Further interactome analysis suggested that the predicted target genes of miR-222-3p constituted a network of signaling pathways.

A few studies have been focusing on miRNA biomarkers for Kawasaki disease. Jia et al. reported that two pairs of serum exosomal miRNAs, including miR-1246/miR-4436b-5p, and miR-197-3p/miR-671-5p, distinguish KD patients from healthy individuals and those with viral infection as candidate diagnostic biomarkers (57). Another study uncovered seven miRNAs were significantly upregulated (hsa-let-7b-5p, hsa-miR-223-3p, hsa-miR-4485, hsa-miR-4644, hsa-miR-4800-5p, hsa-miR-6510-5p, and hsa-miR-765) and three were significantly downregulated (hsa-miR-33b-3p, hsa-miR-4443, and hsa-miR-4515) in plasma of acute KD compared with the healthy controls (58). A similar study claimed that miR-200c and miR-371-5p were elevated in serum in children with Kawasaki disease (59). In our study, hsa-let-7b-5p and hsa-miR-223-3p were slightly downregulated, while miR-200c, miR-197-3p and miR-671-5p was upregulated in KD for 2.09, 2.09 and 2.43 fold, respectively, and the rest of the above mentioned miRNAs were detected in neither KD nor OFI platelet samples. The discrepancy lies in the different background of starting material, indicating specific miRNA expression profile in exosome, plasma and platelets.

There were 35 miRNAs differentially expressed for more than 2 fold, among which miR-222-3p was the top miRNA with highest expression. Previous study revealed that serum miR-221/222 level was significantly elevated in patients with coronary artery disease, suggesting they might be potential diagnostic biomarkers (60). Another important finding is that pathway significance analysis of blood lymphocyte-specific gene markers revealed that T cell receptor signaling pathway is down-regulated in KD, compared to febrile controls (55). Intriguingly, the overlapped miR-222-3p predicted targets were enriched in the T cell receptor signaling pathway and the B cell receptor signaling pathway. Thus, the upregulation of miR-222-3p in KD platelets may partially explain the downregulation due to the crosstalk between platelets and leukocytes. However, more delicate validations are imperative of whether these target genes are indeed regulated by miR-222-3p. These data indicated the involvement of platelet miRNAs in regulation of essential signaling pathways in immune response, which is worthy of further mechanistic investigations.

**Figure 4:**
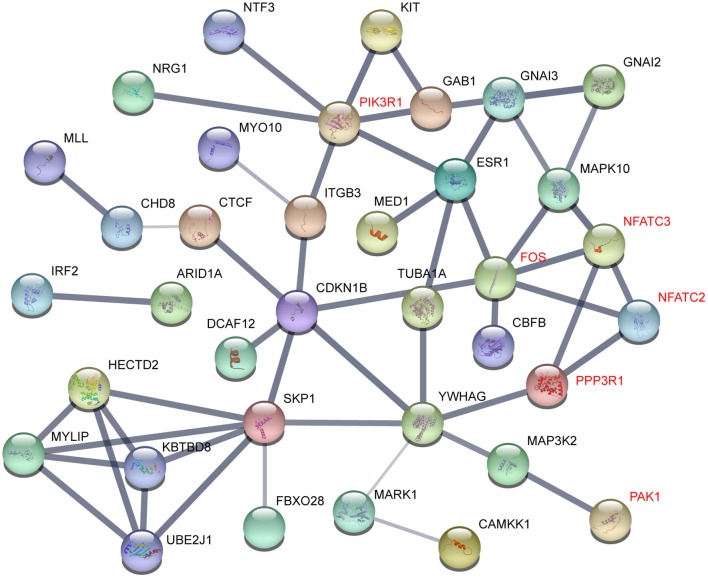
Gene interactions of miR-222-3p predicted targets. Genes related to T cell receptor signaling pathway were denoted red.

To understand the interactome of predicted targets of miR-222-3p, the total 165 targets overlapped by the three prediction tools were used to construct the interaction network with STRING (61). The core sub-network was shown with default settings (Figure 4). The genes involved in T and B cell receptor signaling pathway were denoted red, including PIK3R1, FOS, NFATC2, NFATC3, PPP3R1, and PAK1. These genes also interacted with many other factors involved in various signaling pathways, resulting in a network influenced by miR-222-3p.

This study brings the possibility of miR-222-3p as potential diagnostic biomarker for Kawasaki disease. MiR-222 has been reported to participate in the pathogenesis of many inflammatory diseases, including rheumatoid arthritis, atherosclerosis and obesity-related inflammation (62, 63). Comparing to the miRNAs merely enriched by bioinformatics, miR-222-3p was more biological. Besides, even in the KD group, miR-222-3p was lower in those suffering with coronary artery lesion (Supplementary Figure 3), indicating the potential of using miR-222-3p as predictor of CAL. Future work shall be focused on application of this miRNA in clinical diagnosis of KD, such as developing easily handled *in vitro* diagnostic kits and exploring combinatory diagnostic miRNA sets. Besides, it turns out that the KD group patients are prone to have high CRP levels and platelet count at initial diagnosis (Supplementary Figure 2), which has been reported as inflammatory biomarkers for Kawasaki disease (64). Incorporating clinical manifestations such as CRP and/or platelet count with miRNAs as combinatory biomarkers to facilitate diagnosis is worthy of further investigation.

## Human Subjects/Informed Consent Statement

All procedures followed were in accordance with the ethical standards of the responsible committee on human experimentation (institutional and national) and with the Helsinki Declaration of 1975, as revised in 2000. Informed consent was obtained from all patients for being included in the study.

## Animal Studies

No animal studies were carried out by the authors for this article.

## Data Availability

The normalized CPM of miRNAs expressed in the clinical samples are listed in Supplementary Figure 2. The original miRNA sequencing data can be available upon request.

## Ethics Statement

This study was carried out in accordance with the recommendations of guidelines for good clinical practice (GCP) with written informed consent from all subjects. All subjects gave written informed consent in accordance with the Declaration of Helsinki. The protocol was approved by the Children's Hospital of Soochow University ethics committee.

## Author Contributions

WY and DW conceived and planned the study and wrote the paper. JW, BWa, and YH performed the experiments with the help from other authors. YP performed bioinformatics analysis for data from small RNA sequencing experiments. All authors participated in data analysis and figure preparation.

### Conflict of Interest Statement

DW, YP, JW, YH, BWe, and XX were employed by company QIAGEN (Suzhou) Translational Medicine Co. The remaining authors declare that the research was conducted in the absence of any commercial or financial relationships that could be construed as a potential conflict of interest.

## Supplementary Material

Supplementary Figure 1 Patients included in KD and OFI are matched for age and illness day. **(a)** Comparison of age of patients enrolled in each group. **(b)** Comparison of duration of fever before sample collection.

Supplementary Figure 2 Detailed comparison of clinical lab data between KD and OFI samples. WBC, white blood cell; AST, aspartate aminotransferase; CRP, C-reaction protein; PLT, platelet count (at initial diagnosis). Each point represents one sample. ^*^*P* <0.05; ^**^*P* <0.01.

Supplementary Figure 3 MiR-222-3p expression within KD group differentiated with coronary artery lesion. Each point represents one sample.

Supplementary Table 1 Clinical manifestations of samples enrolled in this study.

Supplementary Table 2 CPM of expressed miRNAs.

Supplementary Table 3 Ten most abundant platelet microRNA families expressed in KD samples.

## References

[1] Billoo AG Lone SW Siddiqui S Atiq H. Incomplete Kawaski disease: are we missing it? J Pak Med Assoc. (2009) 59:42–3. 19213377

[2] Kawasaki T Kosaki F Okawa S Shigematsu I Yanagawa H. A new infantile acute febrile mucocutaneous lymph node syndrome (MLNS) prevailing in Japan. Pediatrics. (1974) 54:271–6. 4153258

[3] Rawat A Singh S. Biomarkers for diagnosis of Kawasaki disease. Indian Pediatr. (2015) 52:473–4. 10.1007/s13312-015-0658-2 26121719

[4] Kuo HC Yang KD Chang WC Ger LP Hsieh KS. Kawasaki disease: an update on diagnosis and treatment. Pediatr Neonatol. (2012) 53:4–11. 10.1016/j.pedneo.2011.11.003 22348488

[5] Newburger JW Takahashi M Beiser AS Burns JC Bastian J Chung KJ. A single intravenous infusion of gamma globulin as compared with four infusions in the treatment of acute Kawasaki syndrome. N Engl J Med. (1991) 324:1633–9. 10.1056/NEJM199106063242305 1709446

[6] Wilder MS Palinkas LA Kao AS Bastian JF Turner CL Burns JC. Delayed diagnosis by physicians contributes to the development of coronary artery aneurysms in children with Kawasaki syndrome. Pediatr Infect Dis J. (2007) 26:256–60. 10.1097/01.inf.0000256783.57041.66 17484225 PMC2868827

[7] Rowley AH Gonzalez-Crussi F Gidding SS Duffy CE Shulman ST. Incomplete Kawasaki disease with coronary artery involvement. Prog Clin Biol Res. (1987) 250:357–65. 10.1016/S0022-3476(87)80503-6 3423049

[8] Newburger JW Takahashi M Gerber MA Gewitz MH Tani LY Burns JC. Diagnosis, treatment, and long-term management of Kawasaki disease: a statement for health professionals from the Committee on Rheumatic Fever, Endocarditis, and Kawasaki Disease, Council on Cardiovascular Disease in the Young, American Heart Association. Pediatrics. (2004) 114:1708–33. 10.1542/peds.2004-2182 15574639

[9] Principi N Rigante D Esposito S. The role of infection in Kawasaki syndrome. J Infect. (2013) 67:1–10. 10.1016/j.jinf.2013.04.004 23603251 PMC7132405

[10] Yim D Curtis N Cheung M Burgner D. An update on Kawasaki disease II: clinical features, diagnosis, treatment and outcomes. J Paediatr Child Health. (2013) 49:614–23. 10.1111/jpc.12221 23647873

[11] Harnden A Takahashi M Burgner D. Kawasaki disease. BMJ. (2009) 338:b1514. 10.1136/bmj.b1514 19416993

[12] Sittiwangkul R Pongprot Y Silvilairat S Makonkaewkeyoon K. Clinical spectrum of incomplete Kawasaki disease in Thailand. Paediatr Int Child Health. (2013) 33:176–80. 10.1179/2046905513Y.0000000062 23930731

[13] Yu HR Kuo HC Sheen JM Wang L Lin IC Wang CL. A unique plasma proteomic profiling with imbalanced fibrinogen cascade in patients with Kawasaki disease. Pediatr Allergy Immunol. (2009) 20:699–707. 10.1111/j.1399-3038.2008.00844.x 19170925

[14] Yu HR Kuo HC Huang EY Liang CD Hwang KP Lin IC. Plasma clusterin levels in predicting the occurrence of coronary artery lesions in patients with Kawasaki disease. Pediatr Cardiol. (2010) 31:1151–6. 10.1007/s00246-010-9769-7 20711835

[15] McNeal-Davidson A Fournier A Spigelblatt L Saint-Cyr C Mir TS Nir A. Value of amino-terminal pro B-natriuretic peptide in diagnosing Kawasaki disease. Pediatr Int. (2012) 54:627–33. 10.1111/j.1442-200X.2012.03609.x 22414326

[16] Dahdah N Siles A Fournier A Cousineau J Delvin E Saint-Cyr C. Natriuretic peptide as an adjunctive diagnostic test in the acute phase of Kawasaki disease. Pediatr Cardiol. (2009) 30:810–7. 10.1007/s00246-009-9441-2 19365652

[17] Kawamura T Wago M Kawaguchi H Tahara M Yuge M. Plasma brain natriuretic peptide concentrations in patients with Kawasaki disease. Pediatr Int. (2000) 42:241–8. 10.1046/j.1442-200x.2000.01225.x 10881579

[18] Kim M Kim K. Elevation of cardiac troponin I in the acute stage of Kawasaki disease. Pediatr Cardiol. (1999) 20:184–8. 10.1007/s002469900437 10089241

[19] Yu X Hirono KI Ichida F Uese K Rui C Watanabe S. Enhanced iNOS expression in leukocytes and circulating endothelial cells is associated with the progression of coronary artery lesions in acute Kawasaki disease. Pediatr Res. (2004) 55:688–94. 10.1203/01.PDR.0000113464.93042.A4 14764920

[20] Ko TM Kuo HC Chang JS Chen SP Liu YM Chen HW. CXCL10/IP-10 is a biomarker and mediator for Kawasaki disease. Circ Res. (2015) 116:876–83. 10.1161/CIRCRESAHA.116.305834 25605650

[21] Parthasarathy P Agarwal A Chawla K Tofighi T Mondal TK. Upcoming biomarkers for the diagnosis of Kawasaki disease: a review. Clin Biochem. (2015) 48:1188–94. 10.1016/j.clinbiochem.2015.02.013 25749557

[22] Kimura Y Yanagimachi M Ino Y Aketagawa M Matsuo M Okayama A. Identification of candidate diagnostic serum biomarkers for Kawasaki disease using proteomic analysis. Sci Rep. (2017) 7:43732. 10.1038/srep43732 28262744 PMC5338030

[23] Zhang G Wang T He Q. How to discover new proteins-translatome profiling. Sci China Life Sci. (2014) 57:358–60. 10.1007/s11427-014-4618-1 24532458

[24] Sabrkhany S Kuijpers MJ Verheul HM Griffioen AW Egbrink MG. Platelets: an unexploited data source in biomarker research. Lancet Haematol. (2015) 2:e512–3. 10.1016/S2352-3026(15)00225-2 26686404

[25] Best MG Vancura A Wurdinger T. Platelet RNA as a circulating biomarker trove for cancer diagnostics. J Thromb Haemost. (2017) 15:1295–306. 10.1111/jth.13720 28671345

[26] Husemann Y Geigl JB Schubert F Musiani P Meyer M Burghart E. Systemic spread is an early step in breast cancer. Cancer Cell. (2008) 13:58–68. 10.1016/j.ccr.2007.12.003 18167340

[27] Liu X Sempere LF Ouyang H Memoli VA Andrew AS Luo Y. MicroRNA-31 functions as an oncogenic microRNA in mouse and human lung cancer cells by repressing specific tumor suppressors. J Clin Invest. (2010) 120:1298–309. 10.1172/JCI39566 20237410 PMC2846041

[28] Sarver AL Li L Subramanian S. MicroRNA miR-183 functions as an oncogene by targeting the transcription factor EGR1 and promoting tumor cell migration. Cancer Res. (2010) 70:9570–80. 10.1158/0008-5472.CAN-10-2074 21118966

[29] Fang L Deng Z Shatseva T Yang J Peng C Du WW. MicroRNA miR-93 promotes tumor growth and angiogenesis by targeting integrin-beta8. Oncogene. (2011) 30:806–21. 10.1038/onc.2010.465 20956944

[30] Diosdado B van de Wiel MA Terhaar Sive Droste JS Mongera S Postma C Meijerink WJ. MiR-17-92 cluster is associated with 13q gain and c-myc expression during colorectal adenoma to adenocarcinoma progression. Br J Cancer. (2009) 101:707–14. 10.1038/sj.bjc.6605037 19672269 PMC2736819

[31] Taganov KD Boldin MP Chang KJ Baltimore D. NF-kappaB-dependent induction of microRNA miR-146, an inhibitor targeted to signaling proteins of innate immune responses. Proc Natl Acad Sci USA. (2006) 103:12481–6. 10.1073/pnas.0605298103 16885212 PMC1567904

[32] Perry MM Moschos SA Williams AE Shepherd NJ Larner-Svensson HM Lindsay MA. Rapid changes in microRNA-146a expression negatively regulate the IL-1beta-induced inflammatory response in human lung alveolar epithelial cells. J Immunol. (2008) 180:5689–98. 10.4049/jimmunol.180.8.5689 18390754 PMC2639646

[33] Moschos SA Williams AE Perry MM Birrell MA Belvisi MG Lindsay MA Expression profiling in vivo demonstrates rapid changes in lung microRNA levels following lipopolysaccharide-induced inflammation but not in the anti-inflammatory action of glucocorticoids. BMC Genomics. (2007) 8:240 10.1186/1471-2164-8-240 17640343 PMC1940008

[34] Lu TX Munitz A Rothenberg ME. MicroRNA-21 is up-regulated in allergic airway inflammation and regulates IL-12p35 expression. J Immunol. (2009) 182:4994–5002. 10.4049/jimmunol.0803560 19342679 PMC4280862

[35] Gu KL Zhang Q Yan Y Li TT Duan FF Hao J. Pluripotency-associated miR-290/302 family of microRNAs promote the dismantling of naive pluripotency. Cell Res. (2016) 26:350–66. 10.1038/cr.2016.2 26742694 PMC4783473

[36] Wang Y Melton C Li YP Shenoy A Zhang XX Subramanyam D. miR-294/miR-302 promotes proliferation, suppresses G1-S restriction point, and inhibits ESC differentiation through separable mechanisms. Cell Rep. (2013) 4:99–109. 10.1016/j.celrep.2013.05.027 23831024 PMC3740202

[37] Ma Y Yao N Liu G Dong L Liu Y Zhang M. Functional screen reveals essential roles of miR-27a/24 in differentiation of embryonic stem cells. EMBO J. (2015) 34:361–78. 10.15252/embj.201489957 25519956 PMC4339122

[38] Xu N Papagiannakopoulos T Pan G Thomson JA Kosik KS. MicroRNA-145 regulates OCT4, SOX2, and KLF4 and represses pluripotency in human embryonic stem cells. Cell. (2009) 137:647–58. 10.1016/j.cell.2009.02.038 19409607

[39] Tay Y Zhang J Thomson AM Lim B Rigoutsos I. MicroRNAs to Nanog, Oct4 and Sox2 coding regions modulate embryonic stem cell differentiation. Nature. (2008) 455:1124–8. 10.1038/nature07299 18806776

[40] Zheng GX Ravi A Calabrese JM Medeiros LA Kirak O Dennis LM. A latent pro-survival function for the mir-290-295 cluster in mouse embryonic stem cells. PLoS Genet. (2011) 7:e1002054. 10.1371/journal.pgen.1002054 21573140 PMC3088722

[41] Yuan K Ai WB Wan LY Tan X Wu JF. The miR-290-295 cluster as multi-faceted players in mouse embryonic stem cells. Cell Biosci. (2017) 7:38. 10.1186/s13578-017-0166-2 28794853 PMC5547456

[42] O'Connell RM Rao DS Chaudhuri AA Baltimore D. Physiological and pathological roles for microRNAs in the immune system. Nat Rev Immunol. (2010) 10:111–22. 10.1038/nri2708 20098459

[43] Lu M Zhang Q Deng M Miao J Guo Y Gao W. An analysis of human microRNA and disease associations. PLoS ONE. (2008) 3:e3420. 10.1371/journal.pone.0003420 18923704 PMC2559869

[44] Bijak M Dzieciol M Rywaniak J Saluk J Zielinska M. Platelets miRNA as a prediction marker of thrombotic episodes. Dis Markers. (2016) 2016:2872507. 10.1155/2016/2872507 28042196 PMC5155104

[45] Ple H Landry P Benham A Coarfa C Gunaratne PH Provost P. The repertoire and features of human platelet microRNAs. PLoS ONE. (2012) 7:e50746. 10.1371/journal.pone.0050746 23226537 PMC3514217

[46] Pontes TB Moreira-Nunes Cde F Maues JH Lamarao LM de Lemos JA Montenegro RC. The miRNA profile of platelets stored in a blood bank and its relation to cellular damage from storage. PLoS ONE. (2015) 10:e0129399. 10.1371/journal.pone.0129399 26121269 PMC4486185

[47] Sunderland N Skroblin P Barwari T Huntley RP Lu R Joshi A. MicroRNA biomarkers and platelet reactivity: the clot thickens. Circ Res. (2017) 120:418–35. 10.1161/CIRCRESAHA.116.309303 28104774

[48] Roy S Sen CK. MiRNA in innate immune responses: novel players in wound inflammation. Physiol Genomics. (2011) 43:557–65. 10.1152/physiolgenomics.00160.2010 21139022 PMC3110889

[49] Snoek J Larochelle H Adams RP Practical bayesian optimization of machine learning algorithms. In: NIPS'12 Proceedings of the 25th International Conference on Neural Information Processing Systems. Advances in neural information processing systems. Lake Tahoe, NV (2012). p. 2951–2959.

[50] Lewis BP Burge CB Bartel DP. Conserved seed pairing, often flanked by adenosines, indicates that thousands of human genes are microRNA targets. Cell. (2005) 120:15–20. 10.1016/j.cell.2004.12.035 15652477

[51] Betel D Koppal A Agius P Sander C Leslie C. Comprehensive modeling of microRNA targets predicts functional non-conserved and non-canonical sites. Genome Biol. (2010) 11:R90. 10.1186/gb-2010-11-8-r90 20799968 PMC2945792

[52] Wang X El Naqa IM. Prediction of both conserved and nonconserved microRNA targets in animals. Bioinformatics. (2008) 24:325–32. 10.1093/bioinformatics/btm595 18048393

[53] Huang da W Sherman BT Lempicki RA. Systematic and integrative analysis of large gene lists using DAVID bioinformatics resources. Nat Protoc. (2009) 4:44–57. 10.1038/nprot.2008.211 19131956

[54] Ikeda K Yamaguchi K Tanaka T Mizuno Y Hijikata A Ohara O. Unique activation status of peripheral blood mononuclear cells at acute phase of Kawasaki disease. Clin Exp Immunol. (2010) 160:246–55. 10.1111/j.1365-2249.2009.04073.x 20015095 PMC2857948

[55] Ling XB Lau K Kanegaye JT Pan Z Peng S Ji J. A diagnostic algorithm combining clinical and molecular data distinguishes Kawasaki disease from other febrile illnesses. BMC Med. (2011) 9:130. 10.1186/1741-7015-9-130 22145762 PMC3251532

[56] Hoang LT Shimizu C Ling L Naim AN Khor CC Tremoulet AH. Global gene expression profiling identifies new therapeutic targets in acute Kawasaki disease. Genome Med. (2014) 6:541. 10.1186/s13073-014-0102-6 25614765 PMC4279699

[57] Jia HL Liu CW Zhang L Xu WJ Gao XJ Bai J. Sets of serum exosomal microRNAs as candidate diagnostic biomarkers for Kawasaki disease. Sci Rep. (2017) 7:44706. 10.1038/srep44706 28317854 PMC5357789

[58] Chen Y Ding YY Ren Y Cao L Xu QQ Sun L. Identification of differentially expressed microRNAs in acute Kawasaki disease. Mol Med Rep. (2018) 17:932–8. 10.3892/mmr.2017.8016 29115644 PMC5780174

[59] Yun KW Lee JY Yun SW Lim IS Choi ES. Elevated serum level of microRNA (miRNA)-200c and miRNA-371-5p in children with Kawasaki disease. Pediatr Cardiol. (2014) 35:745–52. 10.1007/s00246-013-0846-6 24259014

[60] Minami Y Satoh M Maesawa C Takahashi Y Tabuchi T Itoh T Effect of atorvastatin on microRNA 221 / 222 expression in endothelial progenitor cells obtained from patients with coronary artery disease. Eur J Clin Invest. (2009) 39:359–67. 10.1111/j.1365-2362.2009.02110.x 19371267

[61] Szklarczyk D Morris JH Cook H Kuhn M Wyder S Simonovic M. The STRING database in 2017: quality-controlled protein-protein association networks, made broadly accessible. Nucleic Acids Res. (2017) 45:D362–8. 10.1093/nar/gkw937 27924014 PMC5210637

[62] Song J Ouyang Y Che J Li X Zhao Y Yang K. Potential Value of miR-221/222 as diagnostic, prognostic, and therapeutic biomarkers for diseases. Front Immunol. (2017) 8:56. 10.3389/fimmu.2017.00056 28261196 PMC5311065

[63] Ortega FJ Moreno M Mercader JM Moreno-Navarrete JM Fuentes-Batllevell N Sabater M. Inflammation triggers specific microRNA profiles in human adipocytes and macrophages and in their supernatants. Clin Epigenetics. (2015) 7:49. 10.1186/s13148-015-0083-3 25926893 PMC4413548

[64] Simonini G Masi L Giani T Piscitelli E Cimaz R Vierucci S. Osteoprotegerin serum levels in Kawasaki disease: an additional potential marker in predicting children with coronary artery involvement. J Rheumatol. (2005) 32:2233–8. 16265708

