# Supplementary figures and images for: MiR-222-3p in Platelets Serves as a Distinguishing Marker for Early Recognition of Kawasaki Disease
Source: Front Pediatr. 2019 Jun 28;7:237. doi: 10.3389/fped.2019.00237 (PMC6611386; doi:10.3389/fped.2019.00237)

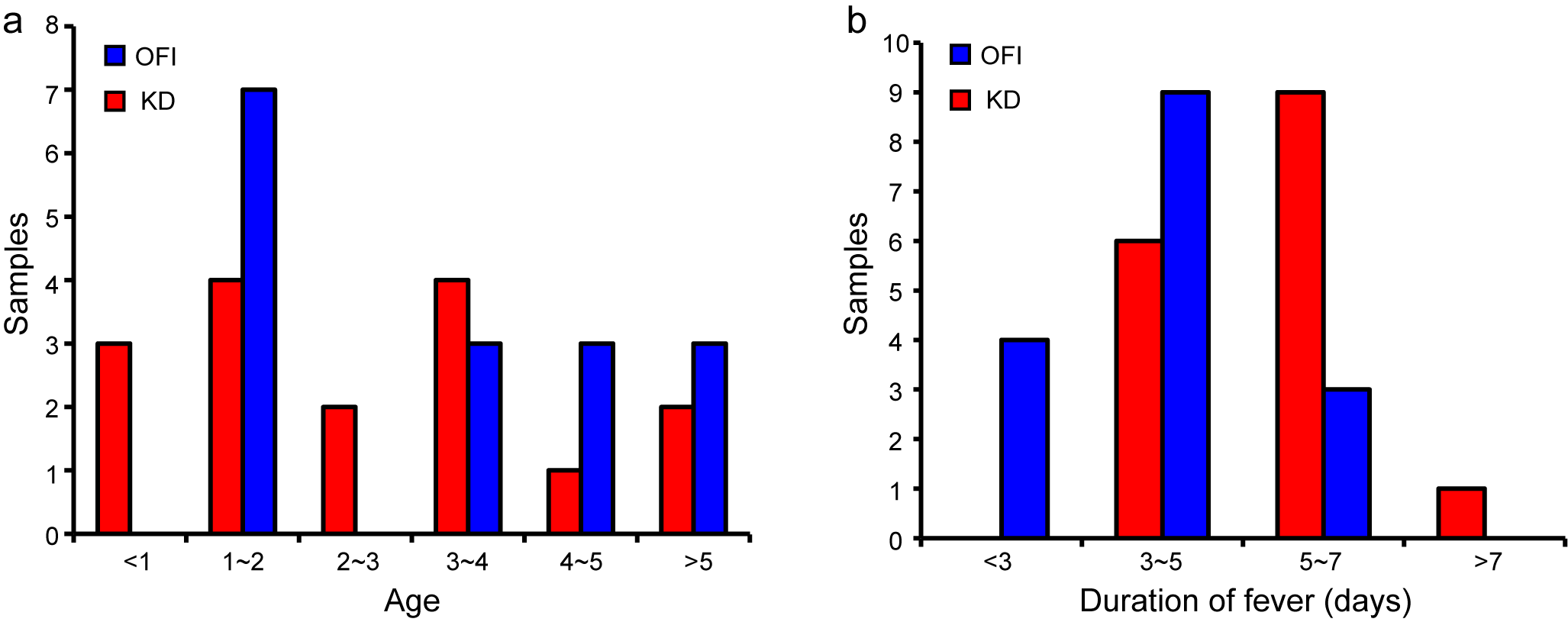

Supplement: Supplementary Figure 1 — Patients included in KD and OFI are matched for age and illness day. (a) Comparison of age of patients enrolled in each group. (b) Comparison of duration of fever before sample collection. [file Image_1.tif]

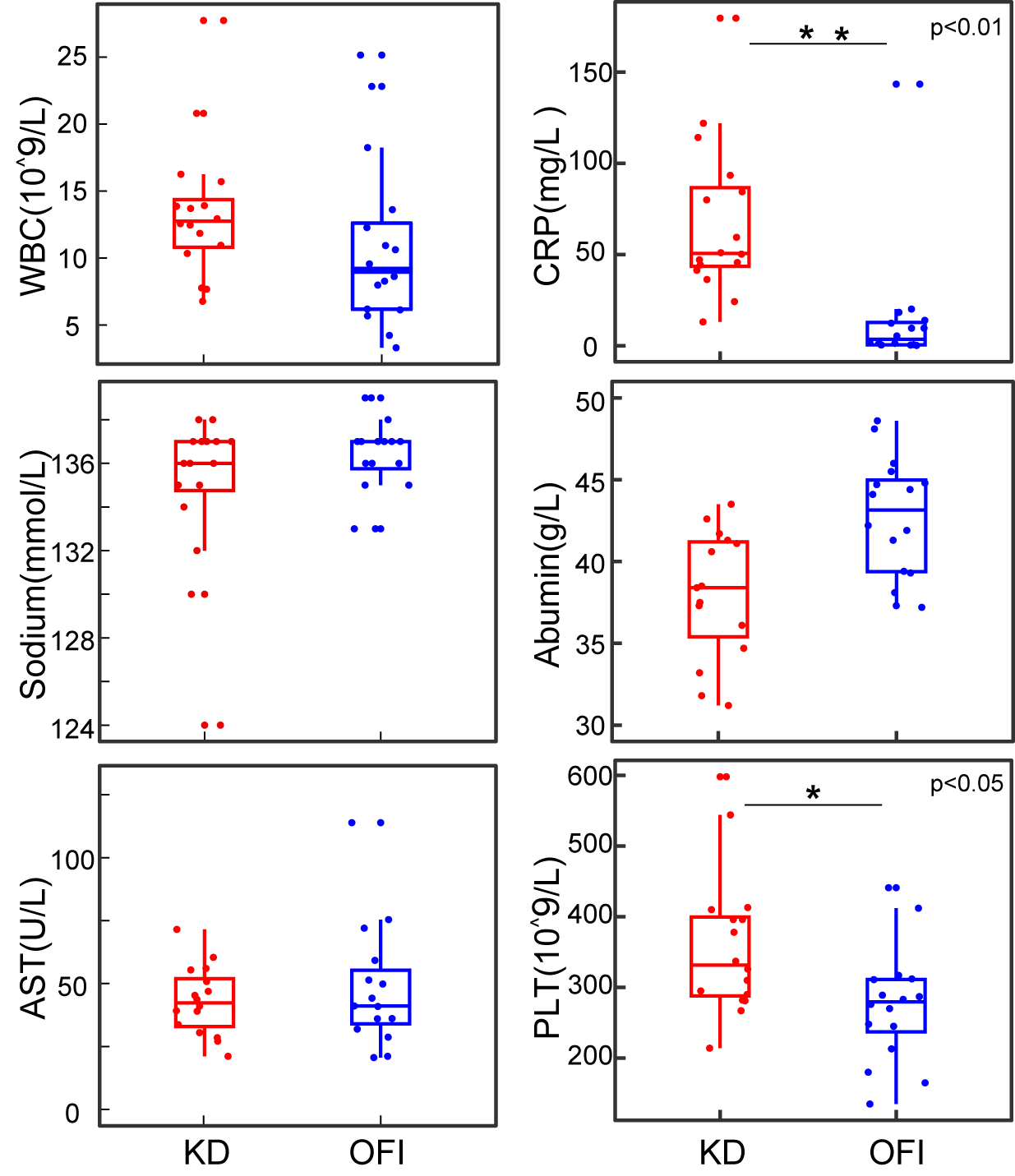

Supplement: Supplementary Figure 2 — Detailed comparison of clinical lab data between KD and OFI samples. WBC, white blood cell; AST, aspartate aminotransferase; CRP, C-reaction protein; PLT, platelet count (at initial diagnosis). Each point represents one sample. *P <0.05; **P <0.01. [file Image_2.TIF]

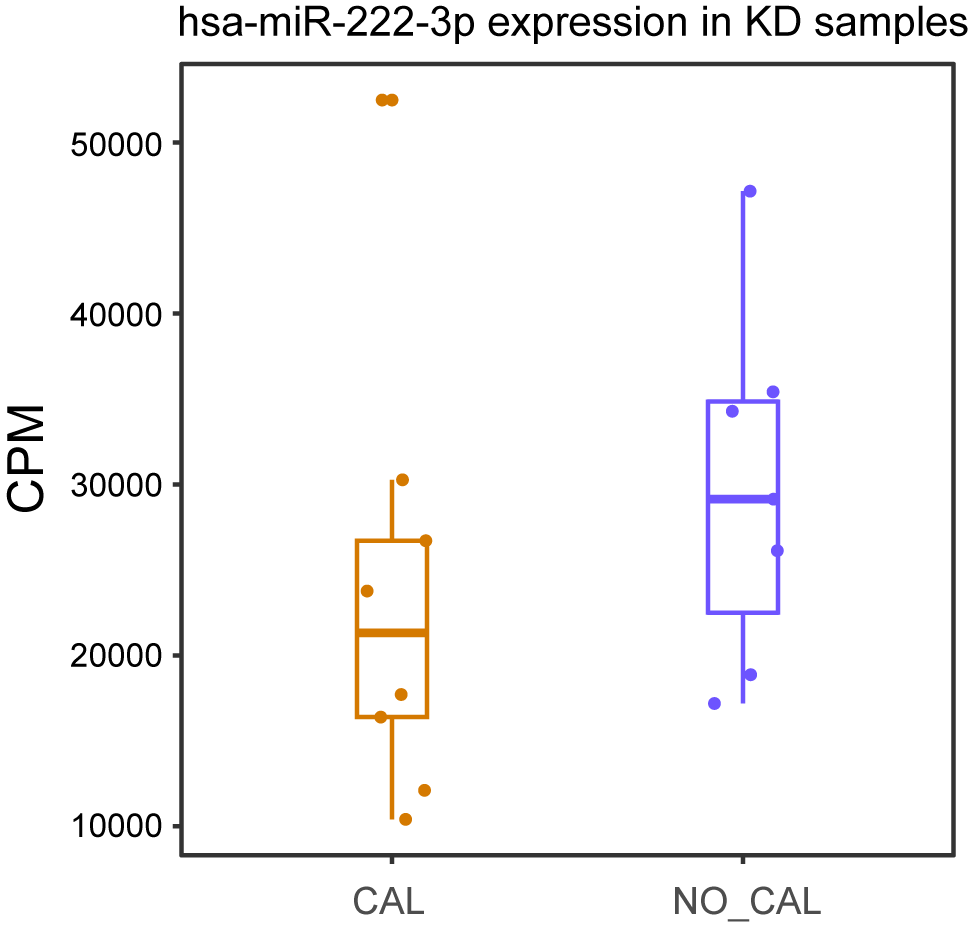

Supplement: Supplementary Figure 3 — MiR-222-3p expression within KD group differentiated with coronary artery lesion. Each point represents one sample. [file Image_3.tif]
